# Slut Shaming in Adolescence: A Violence against Girls and Its Impact on Their Health

**DOI:** 10.3390/ijerph18126657

**Published:** 2021-06-21

**Authors:** Margot Goblet, Fabienne Glowacz

**Affiliations:** Department of Psychology, Université de Liège, 4000 Liège, Belgium; fabienne.glowacz@uliege.be

**Keywords:** slut shaming, violence, adolescence, gender stereotypes, sexual victimization

## Abstract

Slut shaming is defined as the stigmatization of an individual based on his or her appearance, sexual availability, and actual or perceived sexual behavior. It can take place in physical or virtual spaces. The present study questions the impact of this form of sexism in virtual spaces on girls and interrogates the interaction between the values that girls integrate through their life experiences, especially in the family sphere, and slut shaming victimization. We conducted a paper-pencil questionnaire with 605 girls between the ages of 10 and 18 (average age: 15.18 years). Our data confirm the impact of slut shaming on the physical and psychological well-being of young girls as early as adolescence. Second, mediation analyses provide insights into the revictimization and Poly-victimization processes, from childhood adverse experiences to sexist victimization in virtual spaces and their combined impact on the physical and psychic health of girls. Finally, we address prevention strategies and the involvement of socializing institutions in the deconstruction of gender stereotypes.

## 1. Introduction

Slut shaming is defined as the stigmatization of an individual based on of his or her appearance, sexual availability, and actual or perceived sexual behavior and is primarily aimed at women and girls. This stigmatization is reflected in social and relational sanctions, such as rumors, ostracism, or insults, such as “slut” and “fag” [1]. As slut-shaming behaviors can take place on social networks or be exercised via instant messaging or SMS, the Internet and new technologies have made it possible to massively extend the scope of this phenomenon [2]. Our study focuses on slut shaming taking place in virtual spaces during adolescence.

Van Royen [3] found that 18.7% of young people had been called a “slut” on social networks in the six months preceding her study, which concerns 21% of girls aged under 14 (476 girls aged 12 to 18). 10.4% of the participants reported that it had happened several times. According to another study conducted among young Europeans aged 13 to 17 (*n* = 3257), 25% of the participants reported having been victims of sexual rumors in the six months preceding the study. Eighty percent of participants reported having witnessed the use of sexist (“slut”) insults online while 68% had seen homophobic insults online [4].

However, these experiences are often trivialized by young people, and slut shaming appears to be a reality that is difficult to identify and name when it occurs, both in the social and scientific spheres [5]. Is it even a form of violence? At present, studies have not yet explored its impact on the adolescents who are the victims of it nor the experiences over the course of their lives with adults who are important to them who can support them in coping with slut shaming or, on the contrary, make them more vulnerable in virtual spaces. Our study aims to shed light on the previous experiences of victimization of adolescents, especially in the family sphere, interactions with sexist victimization in virtual spaces, and assesses the impact on physical and psychological well-being.

### 1.1. Gender in Adolescence and Slut Shaming as Punishment

In adolescence, puberty modifies the body and relationships with others. Through the changes that their bodies undergo, adolescents see their appearance evolving from childlike to sexual, thereby making them potential objects of desire in the eyes of others. The realization during the first loving and intimate relationships is an unavoidable result of this period of life and induces intense questioning around sexuality and intimacy [6]. This question occurs in the virtual space, too. A space that one might think is disembodied but in which young people—through the photos they post, their connected practices, or the pseudonyms they adopt—create a body in images that they want to be sexualized [7].

Indeed, new technologies offer young people new opportunities to achieve their developmental goals such as identity development and relationships with others, but these new technologies may also create issues around sexuality and intimacy. Sexting, the sending of suggestive messages or images, is one of the contemporary adolescent practices that illustrates this movement [8]. Through sexting, teenagers extend the boundaries of intimacy to the virtual space [9]. However, this practice is not without risk, and current studies testify to the unwanted dissemination, coercion, blackmail, and threats to access these images. Girls are even more likely to suffer the backfire of the production of such content [10]. The double standard sanctions sexting for girls, while it trivializes it for boys, and this sanction can take the form of slut shaming [11].

According to gender schema theory [12], young people learn from childhood to behave as girls or boys through the models they are exposed to. As they grow up, these messages become part of their self-concept and are reflected in their actions, thoughts, and attitudes. Digital technologies add a new dimension to this process, as they provide young people with new, often gender-stereotyped, models of identification and a virtual space in which to perform an “ideal” gender identity [13,14].

This gendered performance is a learning process based on trial and error and is subject to feedback from peers and people who are significant to the adolescent [15]. This feedback can result in social sanctions in the form of slut shaming.

Slut shaming is frequently conceptualized as a form of (cyber)bullying. The consequences of cyberbullying on adolescent development and well-being have been documented in the scientific literature: negative affects; depression and thoughts of suicide; academic difficulties and drop-out; relational issues; alcohol, tobacco, or substance abuse; and polyvictimization [16,17,18,19].

However, framing slut shaming as a form of cyberbullying risks making the gendered dimension of this form of discrimination invisible [17,18,19,20]. “Gender” can be learned [21], particularly through the eyes of peers and the positive or negative feedback they can provide on the gendered performances of young people. Gender is shaped by the socialization contexts of young people, co-constructed by social interactions, and modulated by relational issues, notably with peers and parents [22,23]. This is also true for gendered standards and performances [24,25]. Slut shaming helps to consolidate and perpetuate gender norms and stereotypes and is a form of sexual oppression that is often trivialized [26]. By socially sanctioning behaviors or attitudes that deviate from the established norm for romantic, sexual, or gendered performance, slut shaming reaffirms the dominant codes of normative femininity [2,20,27]. As slut shaming helps to consolidate and perpetuate gender norms [26], we hypothesize that there is a link between gender stereotypes integrated by young people and slut shaming. In other words, slut shaming experiences could contribute to the structuring of certain representations of gender and gender relations, and these representations make young people more vulnerable and less willing to react to this form of gender-based violence. 

Self-presentation that is considered overly sexualized, a multiplicity of sexual partners, and sexual behaviors labelled as “deviant” with respect to the established norms or sexual orientation are likely to lead to slut shaming [7,8,9,10,11,12,13,14,15,16,17,18,19,20,21,22,23,24,25,26,27,28]. Therefore, this form of victimization is essentially gendered. Our first objective is to quantify slut shaming experiences among girl teenagers, starting from early adolescence, and to analyze how it fits into this specific developmental period. This developmental reading invites us to consider the role of the family sphere and the ways in which the childhood experiences of young people allow us to comprehend slut shaming. We pay particular attention to this phenomenon as a form of gendered violence as it relates to certain representations of femininity but also to how differs in this respect from cyberbullying.

### 1.2. Adolescents and Their Parents

Adolescents cannot be comprehended independently of the family sphere in which they live. Although differentiation and empowerment movements are beginning, adolescents are subject to the authority of their parents and are dependent on them. Through their control over their children’s activities and the values that they transmit to them, parents are important agents of socialization in the lives of adolescents and fundamental actors in their identity development and gender construction [23,24].

Moreover, parental practices appear as potential risk or protective factors against the victimization experiences of adolescents. While supervision seems to be a key protective factor [29,30], other studies highlight the importance of parental support, positive parenting practices, and the spontaneous disclosure of activities and relationships, namely parental confidence [31,32].

Regarding the role of parents in the online lives of adolescents, a report [33] indicates that more than half of the young people surveyed in 19 European Union countries believe that their parents can help them when they encounter an unpleasant online experience. Girls and young teens are more likely to ask their parents if they find themselves in trouble online. Duerager and Livingstone [34] found that 89% of parents set rules with their children around giving out personal information online. 82% of parents talk with their children, especially their daughters, about what they do online. 59% stay close by when their child is online. 72% of young people recognize that the measures their parents take with respect to Internet use are useful. However, the authors of this study emphasize that these measures must be adapted to the young person’s age and maturity and that, while measures restricting Internet access diminish opportunities for victimization, they also block access to the resources and positive aspects of virtual spaces. Moreover, young people whose parents use strategies based on communication and information rather than restriction develop better online skills and have a better ability to protect themselves in the virtual space. Finally, some studies [35,36] indicate that parenting practices may have a protective role against cyberbullying. However, it has not been demonstrated that these findings may be relevant to sexist violence in virtual spaces to date. Our study will address the influence of parental intervention, namely parental control and parental disclosure, to prevent gender-based cyberviolence such as slut shaming. 

### 1.3. The Family: A Potential Space for Violent Transactions

While the family environment can mobilize protective and preventive strategies against violence and cyberviolence, it can also make young people vulnerable to and participate in the integration of inappropriate relational models. At the beginning of adolescence, a young person carries a certain amount of baggage, the sum of all the experiences they have had, especially with the adults who are important to them. This baggage endows them with resources but also with fragilities that are likely to modulate their relationship with the world. If the family is a place of growth and development, families can also be a living space where violence and mistreatment are experienced.

Violence can play out between parents and have deleterious effects on the young people who are exposed to it. Several meta-analyses have shown that young people exposed to domestic violence during their childhood are likely to experience difficulties during their development in the short and long term: mood disorders; post-traumatic stress symptoms; substance use (drugs, alcohol, and tobacco); externalized behavioral disorders, such as aggression; internalized behavioral disorders, such as anxiety or depression; suicidal thoughts and actions; academic impairment; and effects on physical well-being and health [37,38,39,40,41,42,43,44]. Exposure to domestic violence may be a risk factor for relational issues and lead to inappropriate or aggressive problem-solving strategies. Indeed, Meyer, Reeves, and Gibbon’s [45] recent study highlighted—among other things—the intergenerational transmission of violence in children that have been exposed to intimate partner violence and the replication of adolescent family violence. Not all the children who have witnessed violence between their parents will be prone to the replication of abusive behavior; however, the existing literature highlights strong associations between past experiences and models of relationships in the future. Bandura’s social learning theory [46], which stresses the role of parents in social learning, including for violent and abusive behaviors, provides us with a conceptual framework to understand those processes. In this perspective, family and extrafamilial socializing influences may also take part in the integration of gender roles and stereotypes as well as in attitudes towards violence. Social learning theory has been shown to apply to the replication of victimization as well [47]. The authors notice that social learning processes are not gender invariant, as previous research demonstrated processes of same-sex imitation in the imitation of aggressive behavior [48].

Moreover, children may themselves be victims of violence at the hands of their parents or caregivers. A meta-analysis including 19 studies and 115,579 participants attests to the impact of physical and sexual violence suffered in childhood and concludes that high levels of depression, anxiety, and distress are reported in adults exposed to childhood sexual and physical abuse [49]. Various meta-analyses confirm the impact that physical abuse can have on the psychological and emotional development and even the long-term health of child victims. For example, young people who suffer abuse from their parents or caregivers are more likely to develop attachment disorders, which can affect their ability to socialize and form relationships with others, have difficulty managing their emotions—they may present more negative emotions—and develop health problems, such as obesity [37,50,51,52]. Other studies [53] identify these youths as being at a higher risk of exhibiting self-mutilating behaviors. Lavi, Katz, Ozer, and Gross [52] put forward several hypotheses as to why children who have experienced maltreatment may have trouble socializing. In particular, they discuss the theory of cognitive processes, demonstrating, for example, how the repetition of violent and negative experiences may contribute to the development of a certain worldview in these young people, who may have a different understanding of reality and social information. This reading of the world could lead to reactions that are less adapted to social cues and an increased vulnerability to violence, in addition to a major risk of depression and anxiety.

Finally, violence may not only be psychological and physical, but also sexual. The family environment is one of the main places where this type of violence against minors occurs. Sexual victimization in childhood is a major traumatic event that can have long-term consequences for an adolescent, which includes risky sexual behaviors such as early sexual initiation, multiple partners, or early pregnancy [54]. Experiences of sexual violence can damage the psychological and social construction of young victims and make them more vulnerable to other forms of victimization. The dynamics of re-victimization following childhood sexual abuse have been identified in scientific literature [55,56,57]. 

The intergenerational transmission of violence theory [58] postulates that experiencing household victimization during childhood may lead to experiencing violence later in life, either as a perpetrator or as a victim. It is possible that these mechanisms of revictimization are transferable to the virtual space [52,55,56]. Our hypothesis is that these traumatic experiences of violence can play a role in making young people vulnerable to victimization in virtual spaces and shape their worldview, particularly with regards to the acceptance of violence and gender stereotypes.

## 2. Materials and Methods

### 2.1. Procedure 

We recruited participants based on the distribution of the population of young people attending secondary education in Belgium Wallonia-Brussels Federation. This study focuses on three different Belgian provinces, thereby allowing access to populations from low, medium, and high socio-economic backgrounds and ensuring a balanced representation of participants from urban and rural areas. We used a purposive sampling. Quotas were established on the basis of statistics compiled by ETNIC on secondary education in the Wallonia-Brussels Federation for the 2015–2016 school year according to three criteria: school year, type of education, and gender. Two hundred and nine schools were contacted by email and telephone. Consent from the parents and the school principals was required. After a pre-test phase, paper questionnaires were collectively administered in 14 French-speaking Belgian secondary schools from March to May 2018. The questionnaires were completed during class hours without the presence of the teachers. They took place in the presence of the researcher who was there to answer any questions and support the completion of the questionnaire. Furthermore, administration of the questionnaire was followed by discussion time with each group, which allowed us to provide the participants with the contact information of helplines and support centers in case of need due to the sensitive nature of the data collected. None of the participants reported discomfort, either during the administration or during the debriefing. Written informed consent was required from the participants and their parents. A random code was attributed to each questionnaire, making it impossible to identify the participants or their schools. Paper questionnaires were kept under lock and only accessible to the research team. The study was conducted according to the guidelines of the Declaration of Helsinki and approved by the Ethics Committee of University of Liège.

605 girls between the ages of 10.25 and 18 took part in this study (average age: 15.18 years).

### 2.2. Measures

#### 2.2.1. Sexual Orientation

Based on the Kinsey Scale [59], we have chosen to measure sexual orientation on a continuum rather than on a heterosexual-bisexual-homosexual categorization. We asked participants to indicate whether they felt attracted to boys only, to boys and some girls, to girls and boys, to girls and some boys, or to girls only. This continuum considers a more fluid definition of sexual orientation, as it accounts for varying degrees of sexual orientation towards those of the opposite or same sex gender. Two additional prompts allowed them to indicate “None” or “I don’t know yet”, aiming, on one hand, to include asexual individuals, and on the other hand, to reflect indetermination and sexual questioning, which are part of adolescent sexual development.

#### 2.2.2. Parental Control and Confidence

These dimensions were assessed using five items from the control and confidence sub-dimensions [32] assessed on a Likert scale from 0 to 3. These items assessed parental control over their child (e.g., “My parents know what I do when I am on the computer or on my mobile phone”) as well as the spontaneous adolescent tendency to disclose their activities (e.g., “I tell my parents with whom I spend my free time”; α = 0.69).

#### 2.2.3. Health

Physical well-being was assessed by the A subscale dealing with the somatic symptoms of the General Health Questionnaire [60]. The General Health Questionnaire assesses the extent to which an individual’s physical and psychological health at a given time differs from their usual state through four subscales (somatic symptoms; anxiety/insomnia; social dysfunction; major depression). According to Molina et al. [61], it is one of the most popular and reliable scales to assess physical and psychological disorders. Its psychometric properties have been studied in various countries [62]. Participants were asked to indicate how they had felt in the past month, with items such as “I had headaches”, on a Likert scale from 0 to 3, from “Never” to “Always”. Higher scores indicated poorer health. 

#### 2.2.4. Depression

Depressive affects were addressed through the D subscale of the General Health Questionnaire, on a Likert scale from 0 to 3, from “Never” to “Always” [60]. A higher score indicated more depressive affects (e.g., “I have sometimes thought that life was not worth living”).

#### 2.2.5. Slut Shaming Victimization

Two items were created based on the literature and items used by previous studies, as no validated scale existed at the time that the study was conducted to assess slut shaming victimization. Almazan and Bain [63] asked their participants if they have been hurt or offended by someone who has used derogatory terms (e.g., “slut”). Another study asked the participants how frequently they had been called slut, whore, or similar names in the past six months on SNS while perceiving this as “unwelcome” [3]. The items we created and used for the present study were: “Posting or sending offensive messages of a sexual nature because of dress, make-up or sexual behavior, such as calling them ‘slut’ or ‘faggot’” and “Spreading sexual rumors”. Participants were asked if anyone had ever done this to them online or through new technologies. Each behavior was assigned 0 or 1, depending on whether the answer was negative or positive. 

#### 2.2.6. Exposure to Domestic Violence

To assess this dimension, one item was used: “Have you ever witnessed physical violence between your parents or the adults who care for you (for example, slapping, kicking, punching, etc.)?”. This was scored on a Likert scale from 0 (Never) to 4 (Very often). 

#### 2.2.7. Physical Maltreatment

We asked young people: “Have you ever experienced violence (for example, slapping, kicking, punching, etc.) at the hands of your parents or the adults who care for you?”. This was scored on a Likert scale from 0 (Never) to 4 (Very often).

#### 2.2.8. Childhood Sexual Abuse

Based on previous studies designed to assess sexual victimization and traumatic experiences in childhood [64], we chose to use a single item to assess childhood sexual abuse. We also asked participants to indicate the frequency of such abuse. The item “Have you ever been touched or caressed sexually by an adult?”, scaled on a scale from 0 (Never) to 4 (Very often), was used to assess sexual violence experienced in childhood.

#### 2.2.9. Gender Stereotypes and Acceptance of Violence

Three items were created for the purposes of this study: “It is important for a boy to be virile, macho”, “It is important for a girl to be attractive, seductive”, and “To gain respect, it is sometimes necessary to use force”. The first two items measured adherence to male and female gender stereotypes, while the third measured acceptance of violence. These items were scaled on a scale from 0 (Completely false) to 4 (Completely true). They were inspired by previous research on gender stereotypes and attitudes towards violence among Belgian adolescents [65,66].

## 3. Results

### 3.1. Descriptive Statistics

The results showed that 7.2% of participants had already received sexually insulting messages via new technologies (about their clothing, makeup, or sexual behavior), and 10.6% reported having been the target of sexual rumors. In total, 14.85% of the participants had already experienced at least one form of slut shaming, and even before the age of 15, 10.47% reported having already been victims of slut shaming online. Previous research on Belgian adolescent girls aged 12 to 18 found slightly higher rates, as high as 17.4% for the whole sample and 21% for participants before the age of 15 [3]. These differences can be explained by the diversity of the items used to measure slut shaming victimization. Comparing our results with other studies is difficult, as slut shaming has not always been contextualized in virtual spaces by previous research. Our results demonstrate that young girls are exposed to gender-based violence in virtual spaces from the beginning of adolescence.

Regarding sexual orientation, 79.83% of girls said they were only attracted to boys. As shown in Table 1, we find very different rates of victimization across the groups, with up to 57.14% of the youth in the bisexual category reporting having experienced slut shaming (12% for heterosexual-only participants). However, given this large majority of only heterosexual youth and the small number of youths in some of the other proposed categories (*n* = 7 to *n* = 35), the data do not allow us to conduct comparative analyses (see Table 1). 

With regard to traumatic experiences that had taken place in the family environment, 32.06% of the participants had witnessed violence between their parents or caregivers at least once. 39.57% had experienced physical violence at least once from their parents, and 10.74% had experienced sexual touching or fondling by an adult. These results underline the extent to which the family environment can be a site of violence for adolescents.

Stereotypes of masculinity femininity and the legitimacy of the use of violence appear to be present in girls as early as adolescence (see Table 2). A total of 24.3% of the participants support (somewhat or completely) a traditional vision of masculinity, and almost half of the girls (49.4%) agree with the traditional prescriptions of femininity. These figures testify to the prevalence of gender roles and stereotypes from adolescence onwards, particularly the prescriptions aimed at girls. Finally, 39.2% legitimize (somewhat or completely) the use of force in certain circumstances, which gives us an indication of the relationship that these adolescents may have with violence.

### 3.2. Correlation Matrix

We sought to explore the interactions between the experiences adolescents may have had in their home environment, the resources or vulnerabilities they derived from it, and their experiences of slut shaming. In order to do so, we mobilized correlational analyses around control and parental confidence as well as traumatic experiences in childhood. Given that we conceptualize slut shaming as a form of gendered violence linked to stereotypes about femininity but also about violence, these analyses also aim to explore the interactions between sexist experiences and representations. The correlation matrix is shown in Table 3. 

The correlational analyses indicate that there are significant links between childhood victimization experiences and slut shaming victimization. Slut shaming victimization is indeed associated with exposure to domestic violence, physical violence, and sexual childhood abuse. Furthermore, our results show the links between traumatic experiences in childhood and representations of femininity, masculinity, and the use of force: these associations suggest that the experience of violence may contribute to shaping representations of gender relations and the tolerance of violence. More specifically, sexual violence and the exposure to domestic violence are linked to the acceptance of female gender stereotypes while physical violence is associated with stereotypical representations of masculinity.

Slut shaming victimization is not associated with gender stereotypes. However, it is significantly correlated with the acceptance of violence thus legitimizing the use of force that could make girls vulnerable to victimization, perhaps making it more acceptable or tolerable in their eyes because the experiences of victimization contribute to shaping such a worldview.

Finally, control and parental confidence appear to be unrelated to the slut shaming experiences of girls. The involvement of the parents in the activities of their daughters, including in the virtual space, does not appear to be related to slut shaming victimization.

### 3.3. Impact

Research on slut shaming has so far been unable to quantify the damage it causes to girls and women, and for this reason, it has not yet been definitively conceptualized as a form of violence. We are trying to determine whether slut shaming does indeed have an impact on the lives of girls from adolescence onwards. To this end, we conducted regression analyses. Slut shaming predicts health problems (R2 = 0.109; F = 72.23; β = 0.330, and *p* < 0.01) and predicts depressive affect and suicidal thoughts (R2 = 0.086; F = 55.814; β = 0.293, and *p* < 0.01).

### 3.4. Mediation

Based on a total score that includes all forms of traumatic childhood experiences, traumatic experiences in childhood (physical violence, exposure to domestic violence, and childhood sexual abuse) were set as predictors of health problems (Table 4). Mediation analyses confirm that traumatic experiences in childhood predict health problems in adolescence, which has already been demonstrated in previous studies.

Our study highlights an unprecedented result: slut shaming victimization significantly mediates the relationship between traumatic childhood experiences and health problems. This mediation is partially because the direct effect of traumatic experiences on health is still present. In other words, young girls that have faced traumatic experiences during childhood are more likely to have health problems, and this effect is amplified by subsequent victimization experiences, such as slut shaming victimization.

We conducted a second mediation (Table 5) to understand the effect of traumatic childhood experiences and a possible mediating effect of victimization on another aspect of the well-being of adolescent girls, which is depression. The results confirm that experiences of childhood victimization may lead to depression in adolescence. This effect is mediated by slut shaming victimization experiences (partial mediation). 

## 4. Discussion

Adolescence is a period of upheaval in an individual’s relationship to their body, to oneself, and to others. This transformation is not gender-neutral; it takes place under the gaze of peers and is subject to sanctions on their parts, even in virtual spaces. Slut shaming that begins at adolescence thus intervenes in this developmental process and can play a role in shaping the gendered construction of adolescents. How slut shaming experiences influence gender construction, self-esteem, body concerns, and relationships with peers are still avenues for future research. While some studies [3,4] have shown that these mechanisms are present from adolescence onwards, current research has not yet explored the impact of slut shaming as gendered violence on adolescent girls, and particularly on their physical and psychological health, nor how it can affect this specific developmental period. Moreover, the dynamics that are likely to make girls vulnerable to slut shaming are still poorly known: we have chosen to question parental proximity and control and traumatic childhood experiences. 

Our results confirmed that slut shaming affects women and girls: up to 14% of our participants have experienced slut shaming at least once. Gendered processes sanctioning the way femininity is expressed are therefore active from adolescence onwards. The gendered prescriptions to which girls are exposed to in the virtual space remain contradictory and a source of stigmatization [11]. However, can we talk about violence? Indeed, violence is characterized in particular by the presence of an attack on the victim’s integrity, be it on a psychological, physical, sexual, or symbolic level.

In our study, slut shaming has emerged as a predictor of depressive affects and health problems. The impact of slut shaming on the psychological and physical integrity of the young people who are the victims of it invites us to conceptualize it definitively as a form of violence that is likely to affect the development and well-being of adolescents. More than trivial remarks and experiences, gender reminder can be a painful experience for adolescent girls. Slut shaming is hardly identified and recognized as a force of violence, either by social institutions, the scientific world, or the adolescents themselves [5]. It is frequently equated with harassment or cyberstalking, a reading that hides the gendered dimension of this phenomenon and the processes by which it contributes to the reassertion of the gendered order [17,18,19,20]. This specificity of slut shaming as gender-based violence as well as its deleterious effect on girls requires vigilance on the part of adults and professionals.

Various studies [52,55,56] have attested to the fact that the experience of physical or sexual violence in childhood can create psychological fragility in young people who have suffered it, particularly from the standpoint of social adaptability, emotional management, or problem solving, and thus make them more vulnerable to other forms of victimization, sexual or not. Our study allows us to apply these findings to the virtual space and gender-based violence and highlights the mediating role of slut shaming in the relationship between traumatic childhood victimization experiences and poor health and severe depression. In other words, our study confirms the deleterious effect of childhood adverse experiences on the physical and psychological well-being of adolescent girls but also allows us to go one step further and position gender-based violence in the processes of revictimization. This invites us not to trivialize slut shaming victimization but rather to consider the impact it can have on the well-being of young people who have already been weakened by their life history. As a reminder of gender and the place girls are supposed to occupy in the gender order, slut shaming brings women’s vulnerability back into focus. This reminder seems to resonate even more for young people who have been exposed to inappropriate role models in childhood or who have experienced body abuse. Indeed, traumatic victimization experiences are associated with gender stereotypes, arguing that there is a co-construction between the experiences and representations and that both physical and sexual violence contribute to the shaping of gender.

Exposure to domestic violence is in fact closely associated with the experience of slut shaming. A gendered reading may allow us to deepen our understanding of this relationship: the results of the Virage survey [67] show that in France, for example, women are the principle victims of violence between partners and that the perpetrator is most often male. Exposure to a family model in which women are abused and dominated by their partners may contribute to the integration of gender stereotypes and standards and therefore play a role not only in creating social and psychological vulnerabilities in the young people who witness it, but also in shaping their representations of relationships between men and women. Moreover, these experiences are likely to influence attitudes towards violence, including the trivialization of and recourse towards violence in certain situations, as shown in studies [33,37]. Our results suggest that exposure to violent role models may make young people more vulnerable to gendered violence, in both physical and virtual spaces. Correlations between childhood traumatic experiences, gender stereotypes, and the legitimization of the use of force support this hypothesis.

Exposure to domestic violence is in fact closely associated with the experience of slut shaming. A gendered reading may allow us to deepen our understanding of this relationship: the results of the Virage survey [49] show that in France, for example, women are the principle victims of violence between partners and that the perpetrator is most often male. Exposure to a family model in which women are abused and dominated by their partners may contribute to the integration of gender stereotypes and standards and therefore play a role not only in creating social and psychological vulnerabilities in the young people who witness it, but also in shaping their representations of relationships between men and women. Moreover, these experiences are likely to influence attitudes towards violence, including the trivialization of and recourse towards violence in certain situations, as shown in studies [37,40,55]. Our results suggest that exposure to violent role models may make young people more vulnerable to gendered violence, in both physical and virtual spaces. Correlations between childhood traumatic experiences, gender stereotypes, and the legitimization of the use of force support this hypothesis.

Moreover, correlational analyses highlighted that a certain acceptance of violence is associated with slut shaming (“To gain respect, it is sometimes necessary to use force”). According to the theory of cognitive processes [52], youths supporting these representations could be more vulnerable to victimization, particularly of a gendered nature in the virtual space. Alternatively, these experiences of victimization may contribute to shaping a worldview in which violence may be an acceptable response. This last reading supports our hypothesis on revictimization and polyvictimization processes.

The literature posits slut shaming as a form of gendered discrimination, closely related to prescriptions of masculinity and femininity. However, our results did not reveal any interaction between the experience of gender-based violence and the production of gender stereotypes. Yet, slut shaming definitely relates to processes of stigmatization toward youth who deviate from expected gendered models, and this is suggested by our data regarding sexual orientation. Our data suggest that youth who do not identify as exclusively heterosexual may be more likely to experience slut shaming: 12% of only heterosexual girls have already experienced slut shaming online, which concerns 25% of only lesbian girls and up to 57% of bisexual girls. This data allows us to reflect on slut shaming a form of violence intended to regulate the bodies and sexualities of young people through sexist insults, even in the virtual space. They also question its impact as a stigma for non-heterosexual youth. However, these results should be taken with caution as the sample size of the proposed categories (*n* = 7 to 35) does not allow comparative analyses. Experiences of slut shaming victimization among LBTIQ+ youth are an interesting research topic for future studies. 

In summary, our study indicates that it is necessary to extend the notion of polyvictimization to virtual spaces and consider the sexist dimensions as well as how traumatic experiences in childhood contribute to shaping the relationship to violence and gender of adolescents. This is a reminder of the increased vulnerability of girls when it comes to gender-based violence.

Faced with this form of gender-based violence affecting the most vulnerable young people, some guidelines can be proposed for prevention. The family environment and parents or caregivers can assist young people when they encounter difficulties. Parental control and parental confidence (i.e., the spontaneous tendency of young people to tell their parents about their activities and associations) appear in scientific literature as protective factors at various levels during adolescence [29,30,31,32]. Duerager and Livingstone [34] indicate that parents tend to talk more with their daughters than their sons about online activities. However, in our study, parental control or supervision is not related to slut shaming victimization, infirming our hypothesis. This observation invites us to rethink the prevention and support offered to adolescents and to address the nature of the prevention messages that are addressed to girls in particular, especially when it comes to sexuality and gender-based violence. When it comes to new technologies and adolescence, some authors identify a form of “moral panic” that can contribute to the call for and development of restrictions for adolescents [68,69]. Other authors identify similar mechanisms in prevention campaigns around sexuality, which often advocate abstinence rather than the development of skills and empowerment among adolescent girls [70,71]. Our results call for the involvement of young people in a dialogue centered around sexist violence and gender stereotypes. We recommend extending preventive measures beyond the family sphere and mobilizing the transmission and reinforcement of prosocial values with the aim of gradually deconstructing gender stereotypes. Schools in particular are one of the socializing institutions that should be mobilized to carry out this work.

### Limits

Slut shaming is gender-based violence that is difficult to name, and therefore, difficult to assess. To date, there is no validated scale to measure it. One of the limitations of our study is that some measurements had been made with items not previously analysed in a pilot study. We have based our research on the manifestations of slut shaming identified in the scientific literature and have chosen to test the behaviors most frequently identified by the authors [63]. Slut shaming manifestations are polymorphic, and subsequent studies would benefit from exploring them with the aim of constructing a more exhaustive measurement instrument.

Although slut shaming is a form of violence that primarily affects women and girls, exploring the nature of the victimization experiences of boys is another potentially interesting avenue of research. We intend to analyse the male participants’ responses to our questionnaire in a future publication.

Our data provide insights into the specific experiences of LGBTIQ+ youth. However, because we chose to measure sexual orientation along a continuum, we only have small samples in the different modalities. These sample sizes do not allow for robust comparative analyses of heterosexual and non-heterosexual youth, although our data indicate that the latter may be more exposed to gender-based violence in the virtual space. Future studies would benefit from investigating the experiences of these youth in particular.

Finally, scientific literature on the theme of harassment and, more specifically, sexual harassment underlines the importance of being able to determine whether violence occurs between peers of the same or opposite sex in order to identify the gendered dynamics of this process [72]. We did not make this distinction in the present study. Instead, we relied on data from Armstrong, Hamilton, Armstrong, and Seeley [1], who suggest that both girls and boys can be actors in this discourse. However, this question would benefit from verification in subsequent quantitative analyses.

## 5. Conclusions

Our results lead us to conceptualize slut shaming as a form of gender-based violence that takes place from adolescence onwards and is likely to generate physical and psychological suffering among young people. The vulnerability of girls to slut shaming has led us to interrogate how it influences perceptions of gender, and particularly, notions of femininity during adolescence. Adolescents shape their worldviews, abilities, and vulnerabilities related to their life experiences, especially in relation to the violence to which they may have been exposed in their childhood. These links allow us to connect the notions of the re-victimization and the theory of cognitive processes [52]. Finally, we recommend reconsidering the forms of prevention and support offered to girls online. These should, no longer be based on perspectives of control, but rather on perspectives of exchange and dialogue involving schools.

## Figures and Tables

**Table 1:** Sexual orientation and slut shaming victimization rates.

Sexual Orientation	% of the Sample	*n*	Slut Shaming Victimization Rates
Only attracted to boys	79.83	483	12%
Attracted to boys and some girls	5.79	35	25.7%
Attracted to boys and girls	2.31	14	57.14%
Attracted to girls and some boys	1.16	7	14.28%
Attracted to girls only	1.98	12	25%
None	2.15	13	7.69%
I don’t know yet	5.95	36	8.33%

**Table 2:** Frequency table for participants’ opinion on gender stereotypes and the acceptance of violence (%).

Items	Completely False	Somewhat False	Somewhat True	Completely True
“It is important for a boy to be virile, macho”	46.5	29.2	19.7	4.6
“It is important for a girl to be attractive, seductive”	27.4	23.2	38.3	11.1
“To gain respect, it is sometimes necessary to use force”	35.2	25.6	29.1	10.1

**Table 3:** Correlation matrix.

Variables	1.	2.	3.	4.	5.	6.	7.	8.	9.
Slut shaming victimization	1								
Parental confidence	−0.52	1							
Parental control	−0.65	0.314 **	1						
Exposure to domestic violence	0.196 **	−0.153 **	−0.041	1					
Physical violence	0.174 **	−0.180 **	−0.133 **	0.554 **	1				
Childhood sexual abuse	0.217 **	−0.058	−0.005	0.243 **	0.291 **	1			
“It is important for a boy to be virile, macho”	0.039	−0.020	0.045	0.041	0.102 *	0.053	1		
“To gain respect, it is sometimes necessary to use force”.	0.108 **	−0.103 *	0.026	0.070	0.094 *	0.096 *	0.194 **	1	
“It is important for a girl to be attractive, seductive”	0.024	−0.068	−0.083 *	0.113 **	0.052	0.086 *	0.360 **	0.281 **	1

Notes: * *p* < 0.05. ** *p* < 0.01.

**Table 4:** Mediation analyses—Predictors of health problems.

Variables		Effect	(Boot) SE	Z	*p*	(Boot) LLCI	(Boot) ULCI
y = health	Total effect of x on y	0.89	0.08	10.46	0.00	0.72	1.06
x = traumatic experiences	Direct effect of x on y	0.75	0.08	8.83	0.00	0.59	0.92
m = slut shaming victimization	Indirect effect of x on y	0.14	0.04			0.07	0.22

Note: SE = standard error; LLCI = lower limit of confidence interval; ULCI = upper limit of confidence interval. Observations with missing values were removed from the analysis.

**Table 5:** Mediation analyses—Predictors of depression.

Variables		Effect	(Boot) SE	Z	*p*	(Boot) LLCI	(Boot) ULCI
y = depression	Total effect of x on y	0.32	0.03	10.30	0.00	0.26	0.38
x = traumatic experiences	Direct effect of x on y	0.28	0.03	8.83	0.00	0.22	0.34
m = slut shaming victimization	Indirect effect of x on y	0.04	0.01			0.02	0.08

Note: SE = standard error; LLCI = lower limit of confidence interval; ULCI = upper limit of confidence interval.

## Data Availability

The data presented in this study are available on request from the corresponding author. The data are not publicly available due to ethical restriction.
